# Detecting Both the Mass and Position of an Accreted Particle by a Micro/Nano-Mechanical Resonator Sensor

**DOI:** 10.3390/s140916296

**Published:** 2014-09-02

**Authors:** Yin Zhang, Yun Liu

**Affiliations:** 1 State Key Laboratory of Nonlinear Mechanics (LNM), Institute of Mechanics, Chinese Academy of Sciences, Beijing 100190, China; 2 Faculty of Information and Automation, Kunming University of Science and Technology, Kunming 650051, China; E-Mail: liuyunkm@126.com

**Keywords:** inverse problem, mass sensing, resonator sensor, resonant frequency, Galerkin method

## Abstract

In the application of a micro-/nano-mechanical resonator, the position of an accreted particle and the resonant frequencies are measured by two different physical systems. Detecting the particle position sometimes can be extremely difficult or even impossible, especially when the particle is as small as an atom or a molecule. Using the resonant frequencies to determine the mass and position of an accreted particle formulates an inverse problem. The Dirac delta function and Galerkin method are used to model and formulate an eigenvalue problem of a beam with an accreted particle. An approximate method is proposed by ignoring the off-diagonal elements of the eigenvalue matrix. Based on the approximate method, the mass and position of an accreted particle can be decoupled and uniquely determined by measuring at most three resonant frequencies. The approximate method is demonstrated to be very accurate when the particle mass is small, which is the application scenario for much of the mass sensing of micro-/nano-mechanical resonators. By solving the inverse problem, the position measurement becomes unnecessary, which is of some help to the mass sensing application of a micro-/nano-mechanical resonator by reducing two measurement systems to one. How to apply the method to the general scenario of multiple accreted particles is also discussed.

## 1. Introduction

In proteomics, mass spectrometry plays an important role in identifying protein species with small sample volume [1,2]. Characterizing the proteome at the single-cell or single-molecule level can accelerate the identification of protein, disease biomarkers and, thus, new drug development [3,4]. However, conventional mass spectrometry typically involves the measurement of around 10^8^ molecules [5], and mass sensing of a cell or a molecule is thus often beyond its limit [4]. Furthermore, because mass spectrometry actually measures the mass-to-charge ratio [1,2], it involves three experimental stages: ionization, separation and detection. For a small and thermostable compound, there is no effective ionizing technique, which is a major restriction for the mass spectrometry application [2]. Ionization may cause structural changes in a protein [5] or damage fragile biological macromolecules [6]. The mass sensing mechanism of a mechanical resonator is the resonant frequency, which shifts when a mass is loaded. The first two stages of ionization and separation are unnecessary for a mechanical resonator, because it can work with neutral species [7]. The motion of a mechanical resonator can be recorded by a single-electron transistor [8,9], the interferometric technique [10], a photodiode [11–13] or a piezoresistive readout [14], from which the resonant frequencies are found. By scaling down in size and selecting materials with high Young's modulus-to-density ratios, the resonant frequency of a mechanical resonator increases, which leads to a higher sensitivity of mass sensing [6]. Because of the high mass sensitivity and frequency stability, a micro-/nano-mechanical resonator provides a label-free, high-throughput and rapid detection of biological and chemical molecules [15]. The ultimate mass sensing limit for a micro-/nano-mechanical resonator is imposed by thermodynamic fluctuation, which has been theoretically proven to be well below one Dalton (1 Dalton ≈ 1.65 × 10^−24^ g is approximately the mass of a proton or a neutron) [16]. The holy grail of achieving the sensitivity to detect the mass of one Dalton has been a major driving force for the recent development of a mechanical resonator sensor. The sensitivity of micro-/nano-mechanical resonators has been improving roughly about an order of magnitude per year for several years [4]. The micro-/nano-mechanical resonator sensors, which can detect the adsorption of a protein [4], a biomolecule [15], a cell [17], a virus [18] and an atom [9,19], have been developed. The holy grail has recently been obtained by Chaste *et al.* [20], who developed a carbon nanotube-based resonator capable of detecting one Dalton mass. Although the achievements are very impressive, there is a fundamental problem to be solved for the above micro-/nano-mechanical resonator sensors: they can detect the resonant frequency shifts induced by a single atom/molecule, but they cannot measure the mass of individual atoms/molecules [5].

The reason is that the resonant frequency shifts are determined by two convolving coupled parameters: the accreted mass and its position. To detect the position, additional equipment, such as a scanning electron microscope (SEM) [10,21] and optical microscope [13], are needed, which are inconvenient and time-consuming [21]. Besides, SEM has the problem of being applied to non-metallic materials, and the optical imaging method becomes invalid when adsorbate is as small as an atom/molecule or when there is not enough contrast between a cell and solution [15,17]. Tracing the sprayed atoms/molecules/nanoparticles and finding their landing locations on a resonator are also extremely difficult, if not impossible [4]. The uncertainty of the particle position has been a major obstacle of accurately measuring its mass [15]. For a micro-/nano-mechanical resonator sensor, the most important problem to be solved, according to Prof. Knobel [6], is to determine the atom/nanoparticle position. Because a micro-/nano-mechanical resonator sensor measures the (shifts of) resonant frequencies, in a real application, the following inverse problem is encountered: How to use the resonant frequencies to determine both the mass and position of an adsorbate? Dohn *et al.* [21] did the pioneering work of using multiple resonant frequencies to determine the mass and position of an accreted particle by the (approximate) Rayleigh–Ritz method and the error minimization procedure. Hanay *et al.* [5] also used multiple resonant frequencies to determine the masses and positions of multiple accreted proteins by a statistics method. Unlike the above two methods, this study presents a straightforward method to tackle the inverse problem, which shows that the mass and position of an accreted particle can be uniquely determined by measuring two or three resonant frequencies/eigenfrequencies. The accuracy of the inverse problem solving method is also demonstrated and compared with the previous ones. The model and the inverse problem solving method are developed for the case of one accreted particle. In a real application, the scenario of only a single adsorbate landing on a micro-/nano-mechanical resonator is (almost) impossible. There are a number of proteins [4,5], atoms [9,19], molecules [20] and nanoparticles accreted on the surfaces of a micro-/nano-mechanical resonator. How to apply the inverse problem solving method to such scenario is also discussed.

## 2. Model Development

**Figure 1.**
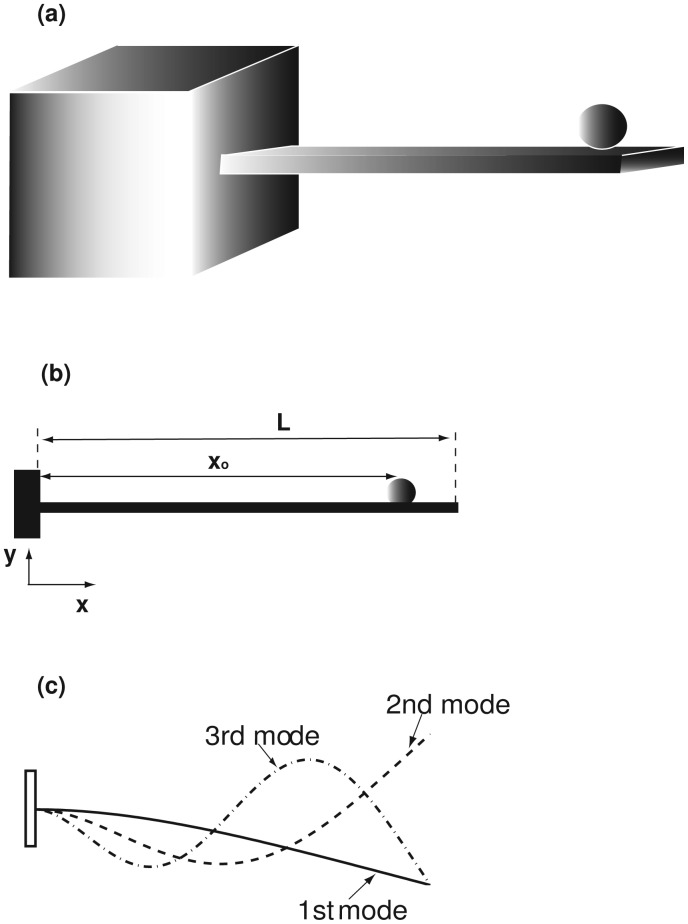
**(a)** Schematic diagram of a cantilever resonator with an accreted particle; **(b)** the coordinate system, the particle position and the beam length; **(c)** the first three modes of a uniform cantilever.

Figure 1a is a schematic of a cantilever beam with an accreted particle. A micro-/nano-mechanical resonator is often modeled as a beam structure [4,5,9,19,20]. For brevity, the beam governing equation is given as follows [22–24]:

(1) $$[m+M_{o}\delta_{D}(x-x_{o})]\frac{\partial^{2}w}{\partial t^{2}}+c\frac{\partial w}{\partial t}+EI\frac{\partial^{4}w}{\partial x^{4}}=0.$$

where *m* is the beam mass per unit length and *m* = *ρbh* (*ρ*, *b* and *h* are the mass density, width and thickness of the beam, respectively). *M*_o_ and *x*_o_ are the mass and position of an accreted particle, as shown in Figure 1b. δ*_D_* is the Dirac delta function, which indicates that the accreted particle is modeled as a concentrated mass [22–24]. *E*, *w* and *c* are the beam Young's modulus, displacement and viscous damping, respectively. *I* is the moment of inertia and *I* = *bh*^3^/12 for a rectangular cross-section beam. There are two major assumptions in Equation (1): (1) the accreted particle only introduces the mass addition effect [25]; and (2) the application of the Dirac delta function assumes that the particle size is extremely small compared with that of the resonator. The accreted particles can change the resonator stiffness, which is mainly induced by two mechanisms: the stiffness of particles [26,27] and surface stress [28,29]. As the particle size is assumed very small, its stiffness can be ignored [26,27]. The presence of surface stress generates both the bending moment and axial force [29,30]; the axial force is responsible for the stiffness change. Surface stress is an important sensing mechanism for many receptor-based sensors [28,31], which is often induced by the receptor-ligand binding. The receptor-ligand binding is both highly sensitive and selective for the identification of an adsorbate/ligand [31]. However, it suffers from poor reproducibility, because of device-to-device variations in the coating. The challenges for developing robust and stable recognition methods through functionalized coatings (*i.e.*, the receptor materials) and even interpreting the responses of receptor-based sensors still remain [31]. For a beam with a non-functionalized surfaces, surface stress is often very small and ignored. Because there is no stiffness change, the resonator described by Equation (1) is the so-called mass-loading sensor [25].

By introducing *ξ* = *x*/*L* $\tau =\sqrt{EI/mL^{4}}t$and *W* = *w*/*L* (*L*: beam length) [22,23], Equation (1) is nondimensionalized as follows:

(2) $$[1+\alpha \delta_{D}(\xi -\xi_{o})]\frac{\partial^{2}w}{\partial \tau^{2}}+C\frac{\partial w}{\partial \tau }+\frac{\partial^{4}W}{\partial \xi^{4}}=0.$$

where *α* and *C* are defined as follows:

(3) $$\alpha =\frac{M_{o}}{mL},\;C=c\sqrt{\frac{L^{4}}{EIm}}$$

Physically, *α* is the ratio of the accreted mass to that of a uniform beam; *C* is the dimensionless damping. The Galerkin method is an efficient method for the eigenfrequency computation of a beam with small concentrated masses [22], which assumes the following form for *W* (*ξ*, *τ*):

(4) $$W(\xi ,\tau )=\sum_{j=1}^{N}a_{j}(\tau )\phi_{j}(\xi ),$$

where *N* is the mode number. *φ_j_*(*ξ*) is the *j* -th mode of a uniform cantilever beam [32], and the first three are shown in Figure 1c; *a_j_*(*τ*) is the unknown *j*-th modal amplitude with the presence of the concentrated mass [22]. Substitute Equation (4) into Equation (2), time *φ_i_*(*ξ*) and integrate from 0 to 1; the following governing equations are derived:

(5) $$\text{M}\ddot{\text{q}}+\text{D}\dot{\text{q}}+\text{Kq}=0.$$

Here, ${(}^{\cdot })=\frac{\partial }{\partial \tau }$and **q** is a vector given as **q** = (*a*_1_,*a*_2_, ….., *a_N_*)*^T^* . **M**, **D** and **K** are the N × N matrices of mass, damping and stiffness, respectively, which are given as the following by using the orthonormality property of *φ_j_*(*ξ*) [22,23]:

(6) $$\begin{array}{ccc} {\text{M}}_{\text{ij}}=\delta_{\text{ij}}+\alpha \phi_{\text{i}}\left(\xi_{o}\right)\phi_{\text{j}}\left(\xi_{o}\right), & {\text{D}}_{\text{ij}}=\text{C}\delta_{\text{ij}}, & {\text{K}}_{\text{ij}}=\kappa_{\text{j}}^{4}\delta_{\text{ij}} \end{array}.$$

where *δ_ij_* is the Kronecker delta function; $\kappa_{j}^{2}$is the j-th (dimensionless) eigenfrequency of a uniform undamped beam, and the first three $\kappa_{j}^{2}$of a cantilever beam are given as follows [32]:

(7) $$\begin{array}{ccc} \kappa_{1}^{2}=1.875^{2}=3.516, & \kappa_{2}^{2}=4.694^{2}=22.034, & \kappa_{3}^{2}=7.855^{2}=61.697 \end{array}.$$

Equation (5) is a damped nongyroscopic system and needs to be rewritten in the following form to formulate an eigenvalue problem [33]:

(8) $$\text{M}*\dot{\text{x}}+\text{K}*\text{x}=0.$$

where **x** = (**q̇**, **q**) is a 2*N* vector; M^*^ and K^*^ are the 2*N* × 2*N* matrices defined as follows [33]:

(9) $$\text{M}*=\left(\begin{array}{cc} \text{M} & 0\\ 0 & -\text{K} \end{array}\right),\;\text{K}*=\left(\begin{array}{cc} \text{D} & \text{K}\\ \text{K} & 0 \end{array}\right)$$

By letting **x**(*τ*)= *e^iωτ^***X**, Equation (8) formulates a standard eigenvalue problem of **AX** = *iω***X** with **A** = − (M^*^)^−1^K^*^ [33]. For Equation (8) to work, α and *ξ_o_* must be supplied. Solving the eigenvalue problem of Equation (8) is not an easy task. As far as the concentrated mass is not located at the fixed end or node (*i.e.*, *φ_j_*(*ξ_o_*) = 0), there are off-diagonal elements, and obtaining the analytical solution to Equation (8) is extremely difficult, if not impossible. With the presence of damping, the eigenvalue of *ω* is a complex variable of *ω* = *R* + *iI*. The real part (*R*) is the eigenfrequency, and the imaginary part (*I*) indicates the stability of the system [34]. Equation (8) yields 2*N* accurate eigenvalues of *R* and *I*.

If the concentrated mass is small, the following approximate analytical solution can be derived. By assuming $W=\sum_{j=1}^{N}b_{j}e^{i\omega \tau }(\tau )\phi_{j}(\xi )(\omega =R+iI)$and repeating the same procedure of the Galerkin method above, the following equation is derived:

(10) GB=0.

where B = (*b*_1_,*b*_2_, ….*b_N_*)*^T^* and ${\text{G}}_{\text{ij}}={\text{G}}_{\text{ij}}^{\text{r}}+i{\text{G}}_{\text{ij}}^{\text{i}}$with ${\text{G}}_{\text{ij}}^{\text{r}}=\kappa_{j}^{4}\delta_{ij}-IC\delta_{ij}-\left(R^{2}-I^{2}\right)-\left[\delta_{ij}+\alpha \phi_{i}(\xi_{o})\phi_{j}(\xi_{o})\right]$and ${\text{G}}_{\text{ij}}^{i}=RC\delta_{ij}-2RI\left[\delta_{ij}+\alpha \phi_{i}(\xi_{o})\phi_{j}(\xi_{o})\right]$. To have a nontrivial solution of **B**, the determinants of both the real part ${\text{G}}_{\text{ij}}^{\text{r}}$and imaginary part ${\text{G}}_{\text{ij}}^{\text{i}}$must be zero. Therefore, $\text{det}({\text{G}}_{\text{ij}}^{\text{r}})=0$and $\text{det}({\text{G}}_{\text{ij}}^{\text{i}})=0$formulate the eigenvalue problem for *R* and *I*, which can only be solved numerically. If the effect of off-diagonal elements are ignored, *R* and *I* are analytically obtained as follows by setting each diagonal element to zero:

(11) $$R_{j}=\sqrt{\frac{\kappa_{j}^{4}}{1+\alpha \phi_{j}^{2}(\xi_{o})}-\frac{C^{2}}{4{\left[1+\alpha \phi_{j}^{2}(\xi_{o})\right]}^{2}},}$$

(12) $$I_{j}=\frac{C}{2[1+\alpha \phi_{j}^{2}(\xi_{o})]}.$$

$R_{j}=\kappa_{j}^{2}$recovers the j-th eigenfrequency of a uniform undamped beam when *α* =0 and *C* =0 [22]. It is noteworthy to point out that when *C* = 0, $R_{j}=\sqrt{\kappa_{j}^{4}/[1+\alpha \phi_{j}^{2}(\xi_{o})]}$

is the same one obtained by the Rayleigh–Ritz method [21]; *R*_1_ also recovers the one obtained by using the curve fitting method when *ξ_o_* =1 (at which *φ*_1_(1) = 2) [10].

**Figure 2.**
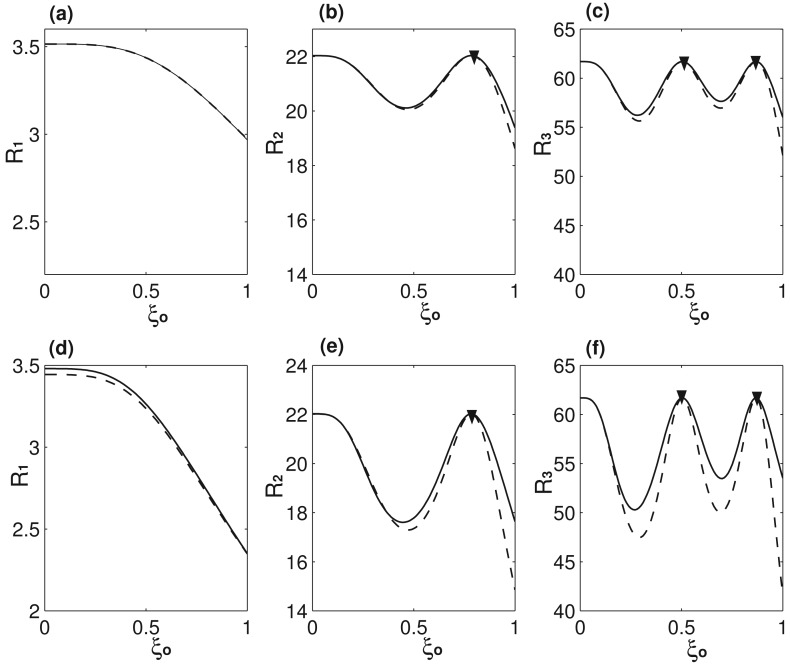
**(a**–**c)** The first, second and third eigenfrequencies with *α* =0.1 and *C* =0.1, respectively; **(d**–**f)** the first, second and third eigenfrequencies with *α* =0.3 and *C* =1, respectively. The solid lines are the results obtained by Equation (8), and dashed lines are those obtained by Equation (11). The solid triangles indicate the nodes.

Figure 2 presents two case studies on the accuracy of Equation (11) as compared with Equation (8) for 0 ≤ *ξ_o_* ≤ 1. Clearly, Equation (11) approximates much better for the case of *α* = 0.1 and *C* = 0.1, than that of *α* = 0.3 and *C* = 1. The reason is simple: Equation (11) ignores the off-diagonal elements, which become more important as *α* increases. The higher mode has higher mass sensitivity, because the effective mass of *α* is larger for higher modes [13], which is also the reason causing the larger error of Equation (11) for higher modes. As noticed in Figure 2, there is no error when a concentrated mass is placed at the node(s). There is no node for the first mode *φ*_1_; *ξ_nd_* = 0.782 is the node of the second mode *φ*_2_; *ξ_nd_* = 0.504 and *ξ_nd_* =0.867 are the two nodes of the third mode *φ*_3_. Mathematically, there are no off-diagonal elements, because *φ_i_*(*ξ_nd_*)=0 (*i* ≥ 2), and physically the effective mass of *α* becomes zero at node(s), which thus has no impact on the eigenfrequency of the corresponding mode.

From Equation (11), we have:

(13) $$\alpha \phi_{j}^{2}(\xi_{o})=\frac{\kappa_{j}^{4}+\sqrt{\kappa_{j}^{8}-R_{j}^{2}C^{2}}-2R_{j}^{2}}{2R_{j}^{2}}.$$

Actually, there are two solutions for $\alpha \phi_{j}^{2}$

As for mass accretion on a mass resonator, $\alpha \phi_{j}^{2}$is positive, as given in the above equation. The other solution of negative $\alpha \phi_{j}^{2}$physically corresponds to a crack formation [34,35] or a vacancy defect [36], which is thus discarded. By setting *j* =1, 2 and 3 and from Equation (13), we have the following:

(14) $$\frac{\phi_{2}^{2}}{\phi_{1}^{2}}(\xi_{o})={\left(\frac{R_{1}}{R_{2}}\right)}^{2}\frac{\kappa_{2}^{4}+\sqrt{\kappa_{2}^{8}-R_{2}^{2}C^{2}}-2R_{2}^{2}}{\kappa_{1}^{4}+\sqrt{\kappa_{1}^{8}-R_{1}^{2}C^{2}}-2R_{1}^{2}},$$

(15) $$\frac{\phi_{3}^{2}}{\phi_{1}^{2}}(\xi_{o})={\left(\frac{R_{1}}{R_{3}}\right)}^{2}\frac{\kappa_{3}^{4}+\sqrt{\kappa_{3}^{8}-R_{3}^{2}C^{2}}-2R_{3}^{2}}{\kappa_{1}^{4}+\sqrt{\kappa_{1}^{8}-R_{1}^{2}C^{2}}-2R_{1}^{2}}.$$

For the convenience of statement, we define left-side functions of the Equations (14) and (15) as $F_{21}\left(\xi_{o}\right)=\phi_{2}^{2}/\phi_{1}^{2}\left(\xi_{o}\right)$ and $F_{31}\left(\xi_{o}\right)=\phi_{3}^{2}/\phi_{1}^{2}\left(\xi_{o}\right)$ ; right-side functions as $S_{21}\left(R_{1},R_{2}\right)={\left(R_{1}/R_{2}\right)}^{2}\left(\kappa_{2}^{4}+\sqrt{k_{2}^{8}-2R_{2}^{2}C^{2}}-2R_{2}^{2}\right)/\left(\kappa_{1}^{4}+\sqrt{k_{1}^{8}-2R_{1}^{2}C^{2}}-2R_{1}^{2}\right)$ and $S_{31}\left(R_{1},R_{3}\right)={\left(R_{1}/R_{3}\right)}^{2}\left(\kappa_{3}^{4}+\sqrt{k_{3}^{8}-2R_{3}^{2}C^{2}}-2R_{3}^{2}\right)/\left(\kappa_{1}^{4}+\sqrt{k_{1}^{8}-2R_{1}^{2}C^{2}}-2R_{1}^{2}\right)$ . One outstanding feature of Equations (14) and (15) is that α does not explicitly appear, whose information is contained in *R*_1_, *R*_2_, *R*_3_. *S*_21_ and *S*_31_ are constants for given *R*_1_, *R*_2_, *R*_3_. *φ*_1_, *φ*_2_ and *φ*_3_ are the given functions of the first, second and third modes of a uniform undamped cantilever beam [32], respectively; the only variable in *F*_21_ and *F*_31_ is *ξ_o_*.

## 3. Results and Discussion

**Figure 3.**
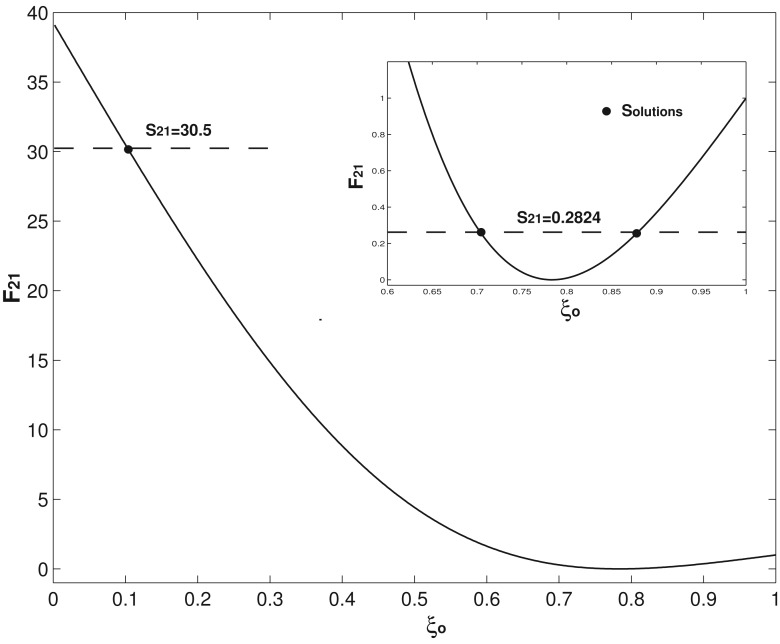
*F*_21_ as a function of *ξ_o_* and the solution of *S*_21_ = 30.5. The inset shows the two solutions when *S*_21_ =0.2824.

**Figure 4.**
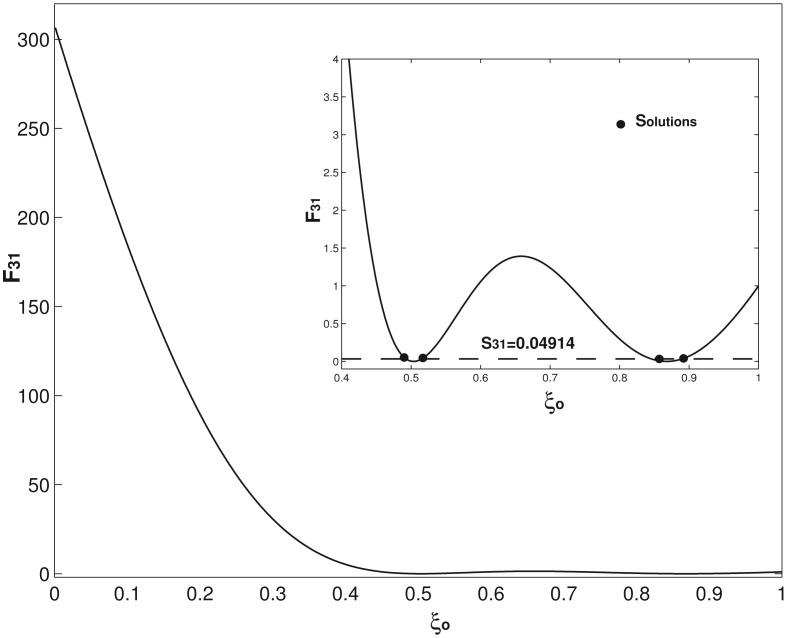
*F*_31_ as a function of *ξ_o_*. The inset shows the four solutions when *S*_31_ =0.04914. There are four *ξ_o_* solutions in 0.451 ≤ *ξ_o_* ≤ 1 when *S*_31_ ≤ 1 and there is only one *ξ_o_* solution in *ξ_o_* < 0.451 when *S*_31_ > 1.

**Figure 5.**
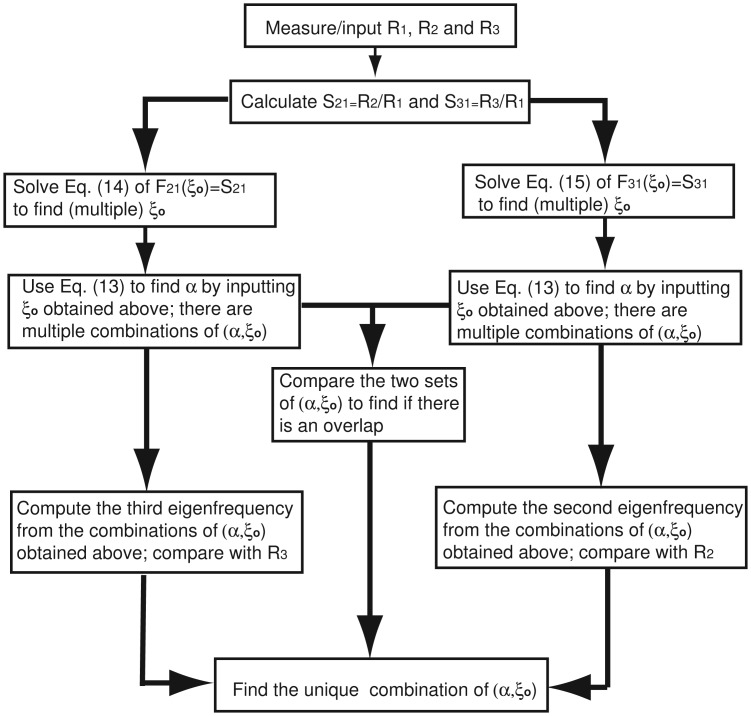
The solution procedure flow chart.

Two examples on how to use Equations (14) and (15) to determine the concentrate mass position and then use Equation (13) to find the corresponding mass are presented. For the first example of (*α*, *ξ_o_*) = (0.1, 0.9) and *C* = 0.1, the first three eigenfrequencies are computed as *R*_1_ =3.086, *R*_2_ = 21.162 and *R*_3_ = 61.25 by using Equation (8). Compared with those of a uniform undamped cantilever presented in Equation (7), all of these three eigenfrequencies decreases because of the concentrated mass and damping. In the application of a mass resonator, *α* and *ξ_o_* are the two unknown parameters to be determined; *R*_1_, *R*_2_, *R*_3_ are obtained by the experimental measurement. Now, suppose the above three *R_i_*s computed by Equation (8) are the experimentally obtained values, which give *S*_21_ = 0.2824 and *S*_31_ =0.04914. Equation (14) is first used, *i.e.*, *F*_21_(*ξ_o_*)= *S*_21_ =0.2824. Equation (14) is nonlinear, and the Newton–Rhapson method is required to solve *ξ_o_*. Figure 3 presents the *F*_21_ – *ξ_o_* relation. As seen in the inset of Figure 3, there are two solutions in 0.636 ≤ *ξ_o_* ≤ 1 and only one solution for *ξ_o_* < 0.636, which physically means that if a concentrated mass locates at any place of *ξ_o_* < 0.636 (or say *S*_21_ > 1), its position can be uniquely determined by Equation (14). *ξ_o_* of *F*_21_(*ξ_o_*) = 0.2824 is solved as *ξ_o_*_1_ = 0.701 and *ξ_o_*_2_ =0.884; substitute these two *ξ_o_*s values into Equation (13), and two corresponding *α*_1_ = 0.213 and *α*_2_ = 0.105 are obtained. Now, we have two possible combinations of (*α*, *ξ_o_*) = (0.213, 0.701) and (0.105, 0.884). Physically, these two combinations generate the same first and second eigenfrequencies, which is the typical scenario encountered in solving an inverse problem [27,29]. To tell which one is the correct one, *F*_31_(*ξ_o_*)= *S*_31_ =0.04914 is needed, which gives four solutions of *ξ_o_*_1_ = 0.4903, *ξ_o_*_2_ = 0.5182, *ξ_o_*_3_ = 0.8409 and *ξ_o_*_4_ = 0.8949. Again, substitute these four *ξ_o_*s into Equation (13) and four corresponding *α*s are obtained as: *α*_1_ = 0.692, *α*_2_ = 0.5727, *α*_3_ = 0.1222 and *α*_4_ = 0.102. There are four combinations, (*α*, *ξ_o_*) = (0.692, 0.4903), (0.5727, 0.5182), (0.1222, 0.8409) and (0.102, 0.8949), and physically, these four combinations generate the same first and third eigenfrequencies. As seen in Figure 4, there are four solutions in 0.451 ≤ *ξ_o_* ≤ 1, and only one in *ξ_o_* < 0.451. Again, this means that if a concentrated mass locates at any place of *ξ_o_* < 0.451 (or say *S*_31_ > 1), its position can be uniquely determined by Equation (15). Now, compare the two combinations obtained by Equation (14) and four combinations obtained by Equation (15); it is not hard to conclude that the only overlapped combination is (*α*, *ξ_o_*)= (0.105, 0.884)/(0.102, 0.8949). Here, the (small) difference between these two combinations is caused by our approximate analytical expression having different errors on different modes, as analyzed above. However, it is still good enough for us to tell which two combinations overlap. Compared with the actual combination of (0.1, 0.9), the accuracy of our method is demonstrated. Alternatively, instead of using $F_{\text{31}}\left(\xi_{o}\right)=S_{\text{31}},F_{\text{32}}\left(\xi_{o}\right)=\phi_{3}^{2}/\phi_{2}^{2}\left(\xi_{o}\right)=S_{\text{32}}$ can also be used to determine (*α*, *ξ_o_*) together with *F*_21_(*ξ_o_*)= *S*_21_. However, keep in mind that *F*_32_ becomes infinite at *ξ_nd_* = 0.782, which is the node of *φ*_2_ and makes the numerical solution more difficult and less accurate. The first three eigenfrequencies of (0.1, 0.9), two combinations obtained by Equation (14) and four combinations obtained by Equation (15) are listed in Table 1. Mathematically, Equation (14) or Equation (15), which, in essence, only uses the information of two (measured) eigenfrequencies, cannot uniquely determine the actual combination of (*α*, *ξ_o_*). Physically, Table 1 contains all of the possible combinations of (*α*, *ξ_o_*) (six in total) determined by two eigenfrequencies; the actual combination of (*α*, *ξ_o_*), which needs to satisfy all three (measured) eigenfrequencies, is among these six possible combinations. There is another effective way to determine the concentrated mass and its position. Equation (14) gives the two combinations with the same first and second eigenfrequencies. If the third eigenfrequency is computed, as seen in Table 1, *R*_3_ = 54.9 for (0.213, 0.701), which deviates significantly from the input/measured value of 61.25 and is thus excluded. The above solution procedure can be summarized by the following flow chart (Figure 5). Measuring the mode shapes, which is frequently done in the structural damage identification [35], can also be used to determine the combination. Although Equation (14) gives two combinations with the same first and second eigenfrequencies, their corresponding mode shapes are different, and mode shape comparison can thus help to determine the right combination. However, the method of the mode shape comparison can be very difficult, especially when the concentrated mass is very small.

In the above example, the concentrated mass of *α* = 0.1 and damping *C* = 0.1 (corresponding to a quality factor of Q ≈ 35) are both relatively large. As demonstrated in the next example, our method achieves a much better accuracy for smaller α and *C*, which is the case in many mass resonator applications [9,10,13,16]. Because the eigenfrequency of a beam is proportional to $\sqrt{EI/\left(mL^{4}\right)}=h/L^{2}\sqrt{E/\rho }$ [23], there are two major methods to enhance the mass resonator sensitivity: (1) to scale down the resonator size [8,16,26] to achieve a larger *h*/*L*^2^; and (2) to use the materials with large *E*/*ρ*, such as graphene [12] and carbon nanotube [9,20]. Both methods result in increasing eigenfrequencies. With a large eigenfrequency, a tiny fractional change in eigenfrequency is still absolutely large enough to be detected [6], and the sensitivity is thus enhanced.

For comparison reasons, the characteristics of Dohn's method [21] is summarized as follows: (1) the damping effect is not included; (2) (at least) four eigenfrequencies are needed to determine the concentrated mass and position; (3) a robust and complex fitting procedure is needed; and (4) more importantly, their method has “inherent shortcomings”, which cannot correctly determine the mass position when *ξ_o_* < 0.2 or *α* < 0.0084 [37]. The second example is presented to show that our method further stands out in the application scenario of *ξ_o_* close to the fixed end with smaller *α* and *C*. (*α*, *ξ_o_*) = (0.0084, 0.1) and *C* =0 [21] are taken and *R*_1_ =3.516 and *R*_2_ = 22.034 are computed by Equation (8), which as inputs give *S*_21_ = 30.5. By using Equation (14), *ξ_o_* = 0.1 is uniquely determined as presented in Figure 3; then substitute this *ξ_o_* = 0.1 value into Equation (13), *α* = 0.0084 is obtained. Now, this method obtains the exact combination of (0.0084, 0.1) and only requires the measurements/inputs of two eigenfrequencies.

In a real application, a micro-/nano-mechanical resonator can be cleaned by passing a large electric current, which generates Joule heating and thus boils off the adsorbates [20]. However, it is (almost) impossible to control the adsorption process to realize the scenario of just one adsorbate. As the model and method are developed for the one particle case, we have to address how the inverse problem solving method can be applied to the general scenario of multiple accreted particles. Theoretically, when the particle number *N* ≥ 2, we can still repeat the above solving procedures with the measurement of up to 3*N* resonant frequencies. However, if *N* is large, the method becomes more complex and much less efficient. Furthermore, experimentally measuring a large number of resonant frequencies is also a big problem, especially for those (very) high modes. Fortunately, we can avoid solving the problem of multiple particles. The reasons are the following three. Firstly, the current state-of-the-art micro-/nano-mechanical resonators are very sensitive, which can detect the shifts of resonant frequencies induced by a single adsorption event. The step-wise decrease of resonant frequency recorded in the experiments indicates the discrete nature of adsorbates arriving at the micro-/nano-mechanical resonator one by one, which is also the hallmark of sensing the individual adsorption events of one protein [4,5], one atom [9,19] and one molecule [20]. By building the histogram of count *versus* frequency shift for the ensemble of sequential single gold atom adsorption, Jensen *et al.* [19] were able to identify with a certain confidence level that the gold atomic mass ranges between 0.1 zg and 1 zg, as compared with the true value of 0.327 zg (1 zg = 10^−21^ g). Proteins in solution often aggregate to form different oligomers, which have different masses [4]. By building the histograms of event probability *versus* frequency shift for the ensembles of sequential single protein adsorption, Naik *et al.* [4] achieved a marvelous result: from the data of 578 individual adsorption events, they can tell that the “nominally pure” protein of bovine serum albumin (BSA) consists of a monomer, dimer, trimer, tetramer, pentamer and their composition. With the help of the second resonant frequency, Hanay *et al.* [5] achieved an even more astonishing result: from the data of 74 individual adsorption events, they can identify 14 different isoforms and their composition of the human IgM antibody. Our inverse problem solving method can do similar work with one datum of one single adsorption event. The underlying rationale is that the above statistics methods [4,5,19] deal with two convolving parameters of an adsorbate: mass and position; therefore, they need tens or hundreds of data to “decouple” these two parameters by assuming certain distribution rules, such as Gaussian; because our method can determine the mass and position of an adsorbate, one adsorption event is enough. Secondly, we need the assumption that the mass of previously adsorbed particles is very small compared with that of a resonator. When there are multiple (unknown) adsorbates, *α* defined in Equation (3) becomes the following:

(16) $$\alpha =\frac{M_{o}}{mL+\sum_{i}^{N}M_{i}}\approx \frac{M_{o}}{mL}$$

where *N* is the (unknown) number of adsorbates and *M_i_* is the corresponding (unknown) mass; *M_o_* is the mass of an incoming particle. The above approximation holds by assuming $\sum_{i}^{N}M_{i}\ll mL$. As discussed above, one great advantage of a micro-/nano-mechanical resonator sensor is to detect the tiny fractional shifts of resonant frequencies, and a very tiny amount of mass can thus be sensed. In Chiu's experiment [9], the mass of the carbon nanotube resonator is around 1000 zg; the adsorbates are the molecules of noble gas, and the atomic masses of xenon and argon are 0.218 zg and 0.066 zg, which corresponds to α =2.18 × 10^−4^ and *α* = 6.6 × 10^−5^, respectively. The mass of previously adsorbed particles has little or no impact on the resonant frequency; the mass and position of a landing adsorbate are mainly or solely responsible for the step-wise resonant frequency drop observed in the experiments, which is also the implicit assumption used in the above statistics methods [4,5,19]. Thirdly, the multiple resonant frequencies induced by an adsorbate can be measured (almost) simultaneously between two individual adsorption events [5]. The resonant frequency of a micro-/nano-mechanical resonator can be as high as one gigahertz or even higher [8,20], and the time required to measured the resonant frequencies is extremely small. On the other hand, the adsorption rate is relatively slow, for example, the argon adsorption rate is 0.09 atom per second [9]. In conjunction with these three conditions, our inverse problem solving method can determine the mass and position of a particle by one single adsorption event, which is more efficient than the statistics approaches [4,5,19]. Furthermore, the statistics approaches are complex, and histogram analysis is time-consuming, which hinders their application in real-time particle mass spectrometry [37]. On the other hand, solving Equations (14) and (15) is much simpler and straightforward, which is capable of real-time analysis.

## 4. Conclusions

An approximate analytical solution for the eigenfrequencies of a mass resonator with a cantilever structure is presented, and its accuracy for small concentrated mass and damping is also demonstrated. The approximate analytical solution is obtained by ignoring the off-diagonal elements of the mass matrix formed by the Galerkin method. The error of the approximate analytical solution becomes large when the concentrated mass or damping is large. The approximate analytical solution can be used to uniquely determine one concentrated mass and its position by measuring at most the first three eigenfrequencies (sometimes only two). The possibility of applying the method to the practical application of the micro-/nano-mechanical resonator mass sensing is discussed. The method can be easily extended to the resonator with the clamped-clamped boundary conditions [8,16,20] by simply changing the mode shape function of *φ_i_*.

## Figures and Tables

**Table 1.** The first three eigenfrequencies for different combinations of (*α*, *ξ_o_*). The combinations are found by Equations (14) and (15), which use the information of two eigenfrequencies.

**(*α*, *ξ****_o_***)/*R****_i_*	***R*_1_**	***R*_2_**	***R*_3_**
(0.1, 0.9)	3.086	21.162	61.25
(0.213, 0.701)	3.084	21.334	54.9
(0.105, 0.884)	3.086	21.353	61.58
(0.692, 0.4903)	3.075	15.264	61.54
(0.5727, 0.5182)	3.077	16.142	61.52
(0.122, 0.8409)	3.085	21.78	61.36
(0.102, 0.8949)	3.085	21.22	61.37
